# Recent insights into SARS‐CoV‐2 omicron variant

**DOI:** 10.1002/rmv.2373

**Published:** 2022-06-04

**Authors:** Severino Jefferson Ribeiro da Silva, Alain Kohl, Lindomar Pena, Keith Pardee

**Affiliations:** ^1^ Department of Pharmaceutical Sciences Leslie Dan Faculty of Pharmacy University of Toronto Toronto Ontario Canada; ^2^ Department of Virology Laboratory of Virology and Experimental Therapy (LAVITE) Aggeu Magalhães Institute (IAM) Oswaldo Cruz Foundation (Fiocruz) Recife Pernambuco Brazil; ^3^ MRC‐University of Glasgow Centre for Virus Research Glasgow UK; ^4^ Department of Mechanical and Industrial Engineering University of Toronto Toronto Ontario Canada

**Keywords:** coronavirus, COVID‐19, omicron, pandemic, SARS‐CoV‐2, variants of concern

## Abstract

The SARS‐CoV‐2 omicron variant (B.1.1.529) was first identified in Botswana and South Africa, and its emergence has been associated with a steep increase in the number of SARS‐CoV‐2 infections. The omicron variant has subsequently spread very rapidly across the world, resulting in the World Health Organization classification as a variant of concern on 26 November 2021. Since its emergence, great efforts have been made by research groups around the world that have rapidly responded to fill our gaps in knowledge for this novel variant. A growing body of data has demonstrated that the omicron variant shows high transmissibility, robust binding to human angiotensin‐converting enzyme 2 receptor, attenuated viral replication, and causes less severe disease in COVID‐19 patients. Further, the variant has high environmental stability, high resistance against most therapeutic antibodies, and partial escape neutralisation by antibodies from convalescent patients or vaccinated individuals. With the pandemic ongoing, there is a need for the distillation of literature from primary research into an accessible format for the community. In this review, we summarise the key discoveries related to the SARS‐CoV‐2 omicron variant, highlighting the gaps in knowledge that guide the field's ongoing and future work.

**Abbreviations**
ACE2: angiotensin‐converting enzyme 2
CDC: centres for disease control and prevention
COVID‐19: coronavirus disease 2019
ED: emergency departments
EM: electron microscopy
FDA: food and drug administration
MERS‐CoV: Middle East respiratory coronavirus
mAbs: monoclonal antibodies
NTD: N‐terminal domain
NT50: 50% neutralisation titer
RBD: receptor‐binding domain
S: Spike
SARS‐CoV: severe acute respiratory coronavirus
SARS‐CoV‐2: severe acute respiratory coronavirus‐2
SGTF: spike gene target failure
TMPRSS2: transmembrane serine protease 2
VSV: vesicular stomatitis virus
VOCs: variants of concern
WHO: world health organization

## 1 INTRODUCTION

Over the last 2 decades, three highly pathogenic coronaviruses have emerged in the human population. The severe acute respiratory coronavirus 2 (SARS‐CoV‐2), the etiological agent of the coronavirus disease 2019 (COVID‐19), has been the latest coronavirus known to emerge from animal reservoirs and cause severe respiratory disease in humans, and was preceded by the severe acute respiratory syndrome coronavirus (SARS‐CoV) and Middle East respiratory syndrome coronavirus (MERS‐CoV) in 2003 and 2012, respectively.^1^, ^2^, ^3^, ^4^ Since its emergence in the human population, SARS‐CoV‐2 has had a catastrophic and unprecedented impact on public health services and the global economy. The rapidly increasing numbers of COVID‐19 prompted World Health Organization (WHO) to declare a pandemic on 11 March 2020^5^ and mobilised public health authorities and scientists around the world to fill the knowledge gaps in clinical practice and basic biology for this unknown virus.

As the COVID‐19 pandemic progressed, SARS‐CoV‐2 has been characterised by the repeated identification of different variants over time and geography: alpha (B.1.1.7) in the United Kingdom, beta (B.1.351) in South Africa, gamma (P.1) in Brazil, and delta in India (B.1.617.2),^6^, ^7^, ^8^, ^9^, ^10^, ^11^, ^12^ which were later designated as variants of concern (VOCs) by the WHO and ushered in a new stage of the pandemic. These emerging variants are the result of natural selection of SARS‐CoV‐2 during serial passage in the host and contain multiple mutations in the receptor‐binding motif, a small 25 amino acid patch at the tip of spike protein that mediates interaction with the human angiotensin‐converting enzyme 2 (ACE2) receptor.^13^, ^14^ Collectively, these mutations in the SARS‐CoV‐2 genome confer fitness advantages, such as increased transmissibility, infectivity, different tropism, modulated virulence, and escape from host immune response induced by vaccination or previous infection.^6^

Approximately 23 months since the first reported case of COVID‐19, the omicron variant (B.1.1.529) was first identified in Botswana and South Africa on 24 November 2021, and then classified as VOC by the WHO on 26 November 2021.^15^, ^16^, ^17^ Since its initial discovery, the omicron variant has outcompeted the delta variant and become the dominant lineage globally with 3,300,603 confirmed cases as of 25 April 2022, thus a clear threat to public health (https://www.gisaid.org/hcov19‐variants/). A growing body of data has demonstrated that the omicron variant is characterised by high transmissibility, robust binding to human ACE2 receptor,^18^, ^19^, ^20^ attenuated viral replication,^21^, ^22^, ^23^, ^24^ causes less severe disease in COVID‐19 patients,^25^, ^26^ and has high environmental stability.^27^ Importantly, the mutations also impart resistance against most therapeutic antibodies,^28^, ^29^, ^30^, ^31^ reduce the ability to induce the immune response in animal models,^32^ and may escape neutralisation by antibodies from convalescent patients or vaccinated individuals.^29^, ^33^, ^34^, ^35^, ^36^, ^37^, ^38^ The rapid spread of the omicron variant has been associated with an abrupt increase in the number of SARS‐CoV‐2 infections, catalysing the fourth wave of the pandemic in many countries worldwide.^15^ With the widespread effort to understand the impact of the SARS‐CoV‐2 omicron variant on COVID‐19 disease, there is a need for the distillation of literature from original research sources into an accessible format for the community.

Based on the scientific knowledge published to date, here, we summarise the latest discoveries of the SARS‐CoV‐2 omicron variant and highlight gaps of knowledge for future investigations. We hope to provide scientific reference for the surveillance and public health measures to counter the SARS‐CoV‐2 omicron variant as the pandemic evolves.

## 2 MUTATIONS IN THE SPIKE PROTEIN OF THE SARS‐CoV‐2 OMICRON VARIANT AND EMERGING SUBVARIANTS

**FIGURE 1:**
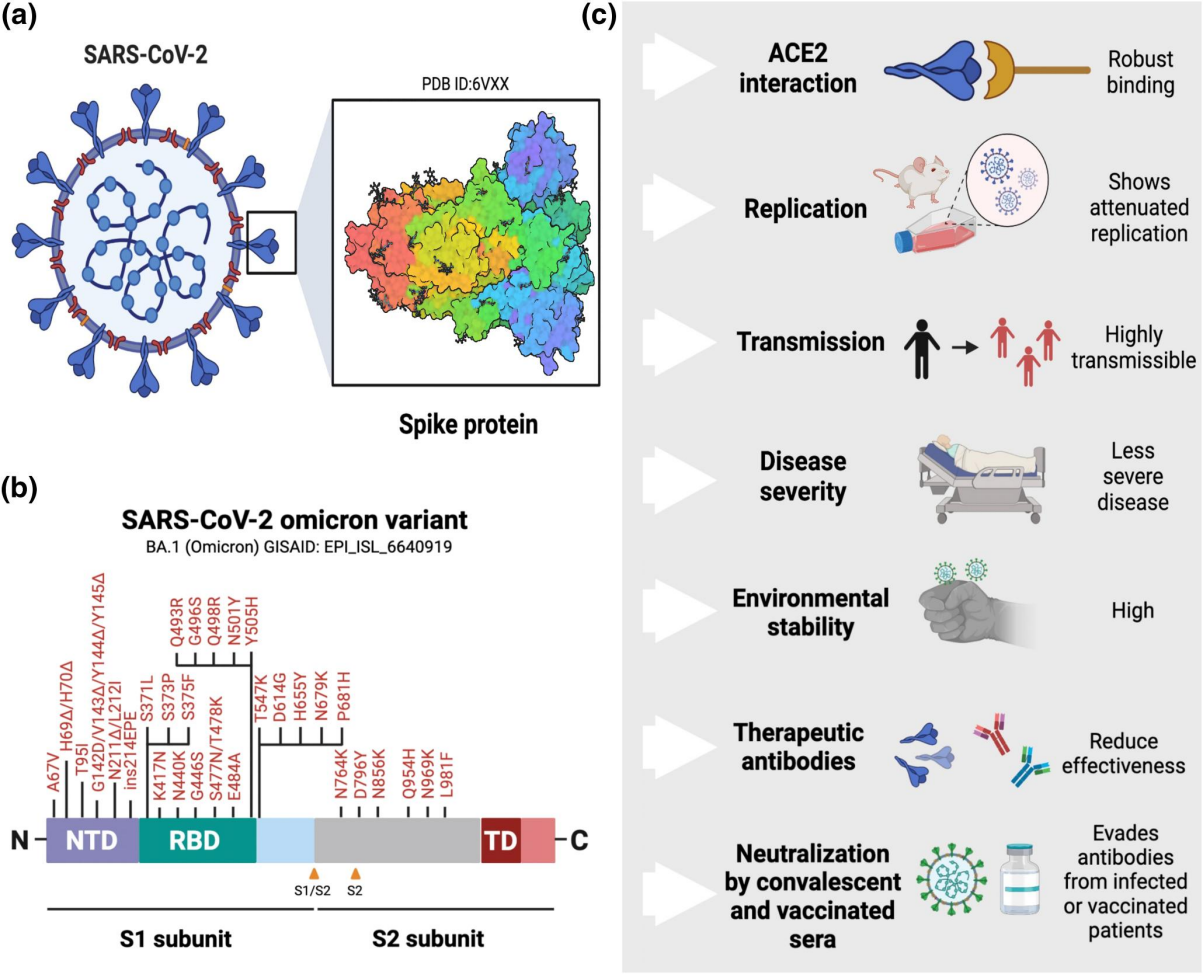
**SARS‐CoV‐2 omicron variant and several characteristics related to this novel variant.** (a) SARS‐CoV‐2 virion and spike protein; (b) the mutations of omicron variant found in the spike protein; (c) the main findings and recent advances related to the omicron variant. The figure was created using Biorender.com

The SARS‐CoV‐2 omicron variant contains a considerable number of mutations in the spike protein compared with previous SARS‐CoV‐2 variants. Mostly concentrated around the receptor‐binding motif, the mutations include 30 amino acid substitutions, deletion of six residues, and insertion of three residues (Figure 1).^19^ Omicron N‐terminal domain of the spike protein harbours 11 mutations, some of which overlap with previously studied SARS‐CoV‐2 lineages, there are mutations (e.g. N211Δ and ins214EPE) that, to date, have only been reported in the SARS‐CoV‐2 omicron variant.^18^, ^19^ Fifteen additional mutations were found in the receptor‐binding domain (RBD) of the spike protein, of which S373P, S371L, S375F and G339D are unique, and nine (map to the ACE2 binding footprint: K417N, G446S, S477N, E484A, Q493R, G496S, Q498R, N501Y and Y505H) were previously known to modulate ACE2 binding and/or immune response.^6^, ^19^ In addition, five mutations were located between the RBD and the S1/S2 site, including the unique mutation T547K and the mutation P681H, which might modulate cleavage at the S1/S2 site.^39^ Within the S2 subunit, six mutations were described.^40^ These changes found in omicron corroborate with the Pango classification, which places the omicron VOC at a substantial distance from all other previous SARS‐CoV‐2 variants.^19^, ^41^

Since its first identification, several subvariants (BA.1.1, BA.2, and BA.3) with strikingly different genetic characteristics have been reported within the omicron variant (https://outbreak.info/). Among them, BA.2 has recently increased in frequency in several regions of the world, suggesting that this subvariant has a selective advantage when compared to other omicron subvariants.^42^ Unlike BA.1, BA.2 contains 8 unique spike alterations and lacks 13 spike alterations found in the original omicron variant (BA.1) and recent findings indicated that this subvariant is antigenically equidistant from the SARS‐CoV‐2 Wuhan virus.^43^, ^44^ Given the current scenario, further studies are needed to understand the consequence of each major emerging subvariant on COVID‐19 disease.

## 3 TRANSMISSION, VIRAL REPLICATION, AND ROBUST INTERACTION WITH ANGIOTENSIN‐CONVERTING ENZYME 2 RECEPTOR

According to the US Centres for Disease Control and Prevention (CDC), omicron is more transmissible (e.g., more easily spread from individual to individual) compared to other SARS‐CoV‐2 variants, including delta.^45^ This was supported by the rapid spread worldwide of the omicron variant in a short time. To confirm this hypothesis, recent reports have investigated the transmission dynamics of the SARS‐CoV‐2 omicron variant through different approaches. Using an artificial intelligence model, it has been suggested that the omicron variant may be over 10 times more contagious than SARS‐CoV‐2 Wuhan virus or about 2.8 times more infectious when compared to the delta variant,^46^ which matched with molecular and epidemiological findings reported by other research teams across the world.^47^, ^48^ Similarly, *in vitro* infection experiments demonstrated that the omicron pseudovirus exhibited higher infection rates that were 4‐fold higher than SARS‐CoV‐2 Wuhan virus and 2‐fold higher than delta variant using 293T‐ACE2 cells or parental 293T cells (without ACE2 receptor).^33^ Taken together, these data strongly suggest differences in transmissibility regarding the omicron variant in comparison with SARS‐CoV‐2 Wuhan virus or other SARS‐CoV‐2 variants.^23^ Despite these early studies, many of the mechanistic details behind the high transmissibility remain to be clarified, along with the real impact of omicron on public health. Comparative transmission studies in relevant animal models such as hamsters and ferrets are warranted.

Virological characteristics of the omicron variant have been also investigated. Two of the most studied factors are the replication competence and cellular tropism of the omicron variant using *in vitro* and *in vivo* models. For instance, Zhao and colleagues investigated the viral replication of the omicron variant and compared it with the delta variant.^22^ This work showed that the omicron variant replicated more slowly than the delta variant in transmembrane serine protease 2 (TMPRSS2)‐overexpressing VeroE6 (VeroE6/TMPRSS2) cells, which provides an interesting way to evaluate the pathway of omicron entry into the host cell.^22^ Moreover, it was found that the omicron variant replicated poorly in the Calu‐3 lung cell line,^22^ which has robust expression of TMPRSS2, a serine protease that has been responsible for S protein priming during SARS‐CoV‐2 entry.^14^ Similarly, a recent report evaluated the replication of the omicron variant using Calu‐3 and the colorectal Caco‐2 cells.^21^ These results revealed that growth of the omicron variant was dramatically attenuated in both cell lineages and was inefficient in TMPRSS2 usage, in comparison to SARS‐CoV‐2 Wuhan virus and other previous variants.^21^ In mice (K18‐hACE2), omicron replication in both the upper and lower respiratory tract of infected animals was considerably lower in comparison to the delta variant.^21^ Taken together, these findings highlight that the omicron variant shows attenuated replication using *in vitro* and *in vivo* models in comparison with SARS‐CoV‐2 Wuhan virus and previous SARS‐CoV‐2 variants.

In a rapidly moving field of study, reported findings do not always align. Other reports have shown opposite outcomes. Hui and colleagues investigated the replication competence and cellular tropism of the Wuhan virus, D614G, alpha, beta, delta and omicron SARS‐CoV‐2 variants in *ex vivo* explant cultures of human bronchus and lung.^49^ The results showed that the omicron variant was able to replicate faster than all other SARS‐CoV‐2 variants in the bronchus but less efficiently in the lung parenchyma,^49^ which the authors suggest likely contributes to higher transmissibility of the omicron variant. In a similar report, Peacock and colleagues showed that the omicron variant replicated faster in human primary nasal epithelial cultures and efficiently uses the endosomal route of entry, more so even than the delta variant.^50^ Moreover, they demonstrated that the omicron variant is capable of efficiently entering cells in a TMPRSS2‐independent route.^50^ This leads to the question of what factors lead to conflicting reports on replication competence? There are a few factors that may be contributing to the differences in phenotypes observed, especially in relation to the selected *in vitro* and *in vivo* models.

Binding affinity of variant spike proteins to the ACE2 from different cell types has been shown to be an important consideration in the infection process. Recent advances using *in silico* and experimental tools have shown that the omicron spike continues to use human ACE2 as its primary receptor, to which it binds more strongly than the original strain from Wuhan and other SARS‐CoV‐2 previous variants.^18^, ^20^, ^50^, ^51^, ^52^, ^53^, ^54^ In one of the earliest studies, Hoffmann and colleagues employed vesicular stomatitis virus (VSV) particles pseudotyped with SARS‐CoV‐2 spike proteins to adequately mimic key characteristics of SARS‐CoV‐2 entry into target cells.^18^ For the analysis of cell tropism, they used the following cell lines: Vero (African green monkey, kidney), 293T (human kidney), A549 (human lung), ACE2 (A549‐ACE2) engineered, Huh‐7 (human liver), Caco‐2 (human colon), and Calu‐3 (human lung) cells.^18^ While subtle differences were observed, these data demonstrated that all cell lines were susceptible to entry driven by all VOCs spike proteins.^18^ Particularly, the omicron spike mediated increased entry into Vero, Huh‐7, and 293T cells.^18^

Supporting this perspective, a recent cryo‐EM structural analysis of the omicron variant spike protein in a complex with human ACE2 revealed new salt bridges and hydrogen bonds formed by mutated residues R493, S496 and R498 in the RBD with ACE2 receptor, suggesting that these alterations appear to compensate other omicron mutations like K417N known to decrease ACE2 binding affinity. The result is a similar biochemical ACE2 binding affinities in comparison to the delta variant.^55^ These findings highlighted that omicron spike bound efficiently to human ACE2 and used it for host‐cell entry, indicating that the mutations in the RBD do not affect ACE2 affinity. Future reverse genetic studies will be key to dissect the impact of these point mutations into SARS‐CoV‐2 biology.

**FIGURE 2:**
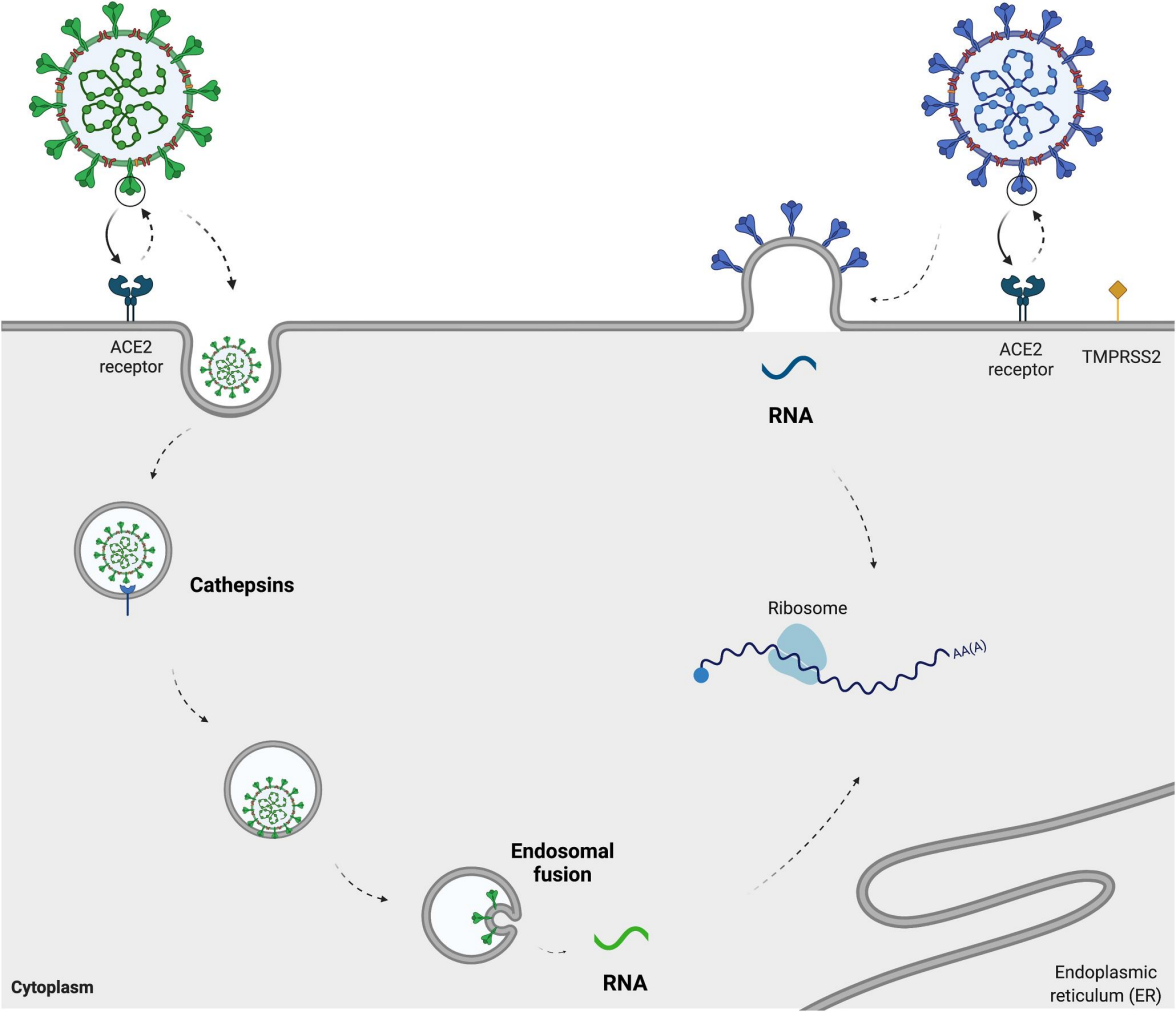
**A schematic illustration of two cell entry pathways that are known to be used by SARS‐CoV‐2.** Recent insights demonstrated that the SARS‐CoV‐2 omicron variant spike enters cells less efficiently by TMPRSS2‐dependent plasma membrane fusion (right) and demonstrates a greater dependency on cell entry via the endocytic pathway (left).^22^, ^24^ ACE2: angiotensin‐converting enzyme 2; TMPRSS2: transmembrane serine protease 2. The figure was created using Biorender.com

Using cell culture experiments, a recent study showed that the omicron demonstrates attenuated fusogenicity (e.g. multistep process, in which the virus binds to the cell membrane) in comparison to delta and an ancestral SARS‐CoV‐2 virus.^23^ Furthermore, it was found that the S protein of omicron is less efficient when cleaved into two subunits,^23^, ^24^ which has been known to facilitate cell‐cell fusion.^56^, ^57^ Recent findings have shown that the omicron variant is more dependent on cathepsins than other previous variants, suggesting that this variant enters cells by a different route.^49^ To explore this relevant question, Meng and colleagues used *in vitro* experiments to demonstrate differential usage of TMPRSS2 as a cofactor for virus entry.^24^ It was found that the omicron spike inefficiently utilises the TMPRSS2 for cell entry via plasma membrane fusion, while demonstrate a greater dependency on cell entry via the endocytic pathway (Figure 2).^24^

## 4 IS THE OMICRON VARIANT LESS VIRULENT THAN PREVIOUS SARS‐CoV‐2 VARIANTS?

Preliminary human clinical data has suggested that the omicron variant was associated with significantly less severe outcomes among infected individuals.^25^, ^26^, ^58^ In one of the earliest reports, the CDC characterised the initial 43 cases attributed to the omicron variant in the US.^59^, ^60^ Among 43 cases with initial follow‐up, only one hospitalisation was reported, which did not prove to be lethal.^59^ In another retrospective cohort study including 14,054 infected patients with the omicron variant from a multicenter, nationwide database in the US from December 2021 until January 2022, the authors compared the outcomes of COVID‐19 disease in paediatric and adult patients before and after the emergence of the omicron variant. It was found that the omicron cohort was associated with significantly less severe outcomes for first‐time infections compared to when the delta variant was predominant in the US.^60^ Interestingly, it was found that the omicron cohort was significantly different when compared to the delta cohort in terms of comorbidities, demographics, and socio‐economic determinants of health.^60^ In children under 5 years old, the overall risks of emergency departments visits and hospitalizations in the omicron cohort were 3.89% and 0.96% respectively, significantly lower than 21.01% and 2.65% for the delta cohort.^60^ Using multivariable logistic regression models, Wolter and colleagues evaluated the clinical severity of the SARS‐CoV‐2 omicron variant in South Africa.^61^ In that study, the authors assessed the disease severity and hospitalizations by comparing individuals with S gene target failure (SGTF), a molecular approach usually applied to detect SARS‐CoV‐2 VOCs such as omicron while awaiting sequencing results, due to the presence of a mutation (69‐70del) in the spike protein of SARS‐CoV‐2 resulting in a deletion of two amino acids at sites 69 (histidine) and 70 (valine).^62^, ^63^ For data analysis, the authors compared SGTF versus non‐SGTF infections diagnosed between 1 October and 30 November 2021. Following this, they evaluated the disease severity by comparing SGTF‐infected individuals diagnosed between 1 October and 30 November 2021, with delta variant‐infected persons diagnosed between 1 April and 9 November 2021.^61^ The study found a significantly reduced odds of hospitalisation among individuals with SGTF versus non‐SGTF infections (delta), while SGTF‐infected individuals had significantly reduced odds of severe illness compared with persons infected previously with the SARS‐CoV‐2 delta variant.^61^ Together, these findings highlight that omicron variant SARS‐CoV‐2 cases with the omicron variant are associated with less severe disease in the human population.

Using *ex vivo* and *in vivo* models, some reports have provided relevant insights into the pathogenicity of the omicron variant.^49^, ^64^ Hui and colleagues compared the replication competence and cellular tropism in *ex vivo* explant cultures of human bronchus and lung.^49^ They showed that the omicron variant replicated faster than Wuhan virus and all other SARS‐CoV‐2 variants (D614G, alpha, beta and delta) in the bronchus but less efficiently in the lung parenchyma.^49^ The authors highlighted that the lower replication competence of omicron variant in human lung may be compatible with reduced severity, although the determinants of severe disease are multifactorial.^49^ Shuai and colleagues investigated the pathogenicity of the omicron variant in K18‐hACE2 mices.^21^ It was found that the replication and pathogenicity of the omicron variant were attenuated in both the upper and lower respiratory tract of infected mice.^21^ In comparison with Wuhan virus and previous SARS‐CoV‐2 variants, the infection by the omicron variant was associated with the least body weight loss and mortality rate.^21^ In another independent *in vivo* study using several mouse lineages (129, C57BL/6, BALB/c and K18‐hACE2 transgenic) and hamsters (wild‐type and hACE2 transgenic), it was found that the omicron variant was linked to a less severe infection in 129, C57BL/6, BALB/c, and K18‐hACE2 transgenic mice when compared with other SARS‐CoV‐2 variants, with limited weight loss and lower viral burden in the upper and lower respiratory tracts.^64^ Similarly, it has been shown that the omicron was also milder in wild‐type and hACE2 transgenic hamsters, demonstrating that this VOC is less virulent to rodents than previous SARS‐CoV‐2 strains.^64^

As the COVID‐19 pandemic evolved, recent insights have suggested that emergent novel omicron subvariants may cause more severe disease than the original omicron variant (BA.1). To assess this question, several research groups have investigated the pathogenicity of emerging omicron subvariants (BA.1.1, BA.2, and BA.3). In one of the earliest reports, an *in vivo* study using Syrian hamsters evaluated the pathogenicity of SARS‐CoV‐2 omicron (R346K, BA.1.1 subvariant) and then compared its cross‐neutralisation and disease characteristics with that of delta variant infection.^65^ Interestingly, the authors showed that the illness characteristics of the BA.1.1 subvariant were found to be similar in comparison with the infection caused by the delta variant in hamsters such as viral replication in the respiratory tract and interstitial pneumonia, indicating that the infection with this subvariant may produce moderate to severe lung disease.^65^ In terms of immunological response, it was also found that the neutralising antibody response against BA.1.1 subvariant could be detected from day 5 and that these antibodies only poorly neutralised previous SARS‐CoV‐2 variants.^65^

The realistic impact of omicron on virulence and mortality in non‐rodent animal models and humans is yet to be answered. A recent mathematical modelling analysis using data from England suggested that omicron does have the potential to cause substantial surges in hospital admissions and deaths in populations with high levels of immunity.^66^ However, observational studies will be useful to confirm this hypothesis over time. Despite omicron appearing to cause less severe infection, there is a fundamental need to understand the mechanisms and pathways by which the omicron variant can impact the COVID‐19 disease severity, especially after the emergence of new omicron subvariants.

## 5 OMICRON FOUND TO HAVE HIGHER ENVIRONMENTAL STABILITY THAN PREVIOUS SARS‐CoV‐2 VARIANTS

Recent advances have been made towards understanding the differences in environmental stability among SARS‐CoV‐2 VOCs. One study investigated the difference in viral environmental stability on plastic and skin surfaces between the SARS‐CoV‐2 Wuhan virus and SARS‐CoV‐2 variants (alpha, beta, delta, and omicron).^27^ It was shown that all SARS‐CoV‐2 variants included in this study exhibited more than two‐fold longer survival than the Wuhan virus and maintained infectivity for more than 16 h on skin surfaces,^27^ with the omicron variant having the highest stability. Thus, these results indicate that the high environmental stability of these SARS‐CoV‐2 variants could increase the risk of contact transmission and contribute to their spread. However, the clinical impact of these data should be taken with caution since the virus spreads from person to person mainly through direct contact or airborne transmission.^67^, ^68^, ^69^

## 6 IMMUNE ESCAPE FROM THE NEUTRALISATION ACTIVITY OF THERAPEUTIC ANTIBODIES

While vaccines remain the most effective approach to prevent SARS‐CoV‐2 infection and disease, the use of therapeutic monoclonal antibodies (mAbs) could potentially benefit certain vulnerable populations before or after exposure to the virus, such as the unvaccinated or recently vaccinated high‐risk persons.^70^ With the emergence of new SARS‐CoV‐2 variants there is an urgent need to investigate the impact of the corresponding mutations on established mAbs and therapeutic antibody products in order to confirm effective strategies for clinical practice. Accordingly, many neutralising mAbs or therapeutics antibody products previously developed for SARS‐CoV‐2 infection are now under evaluation against the omicron variant.^18^, ^28^, ^29^, ^30^, ^31^, ^71^, ^72^, ^73^

To date, this cumulative body of data suggests that the omicron variant is totally or partially resistant against most mAbs or therapeutic antibody products (individually or in combination) under clinical use, or in late stages of clinical development including: casirivimab, bamlanivimab, etesevimab, imdevimab, regdanvimab, etc. This indicates that many of these available mAbs or therapeutic antibody products approved by the Food and Drug Administration (FDA) may be less effective in patients with the omicron SARS‐CoV‐2 variant.^18^, ^28^, ^29^, ^30^, ^71^, ^72^ In contrast, a small proportion of these mAbs or therapeutic antibody products currently available have retained their total or partial potency against the omicron variant. In light of these data, the FDA has revised the authorisations for two monoclonal antibody‐based antivirals (bamlanivimab/etesevimab and casirivimab/imdevimab) to limit their use to only to patients infected with a susceptible strain.^74^ Preliminary experimental data has identified some mAb candidates have retained the potential to effectively neutralise the omicron variant, these include sotrovimab,^18^, ^29^ S2K146,^28^ S2X324,^28^ S2N28,^28^ S2X259,^28^ S2H97,^28^ S309,^30^, ^72^, ^75^ JMB2002,^76^ COV2‐2196 (marketed as tixagevimab),^72^ and COV2‐2130 (marketed as cilgavimab).^72^

## 7 ANTIVIRAL DRUGS FOR TREATMENT OF PATIENTS INFECTED WITH OMICRON VARIANT

In terms of antiviral therapies, recent studies have demonstrated that remdesivir,^24^, ^72^, ^77^ molnupiravir,^24^, ^72^, ^77^, ^78^ nirmatrelvir^77^, ^78^ and PF‐07304814^72^ are effective against infection with the omicron variant, suggesting that these antiviral drugs may be suitable for the treatment of patients with this novel SARS‐CoV‐2 variant. Despite these antiviral options, the therapeutic arsenal available to physicians does appear to be reduced for patients with the omicron variant. To address this gap, *in vitro*, *in vivo* and clinical trials aimed at determining the efficacy of different antiviral drugs against the omicron variant will be of paramount importance to maintain sufficient options for clinical practice.

## 8 IMMUNE ESCAPE FROM THE NEUTRALISATION ACTIVITY AGAINST CONVALESCENT PLASMA

Analysis of convalescent sera from COVID‐19 patients provides relevant insights into antibody longevity and cross‐neutralising activity induced by the SARS‐CoV‐2 spike protein.^79^ A durable neutralising antibody response that provides protection against emerging SARS‐CoV‐2 variants is the best tool in our public health toolbox.^79^ Seeking to understand the consequence of the omicron variant for patients with prior infection, the efficacy of neutralising antibodies from convalescent patients has been analysed in several studies from different parts of the world using selected samples (sera/plasma).^18^, ^80^, ^81^

Using sera/plasma obtained within two months of convalescence from mild or severe COVID‐19 disease collected in Germany during the first wave of the pandemic, it was found that the neutralisation by the omicron spike was 80‐fold less efficiently as compared with the Wuhan virus spike and 44‐fold less efficiently as compared with delta spike.^18^ In another similar study using specimens obtained at approximately 1 month and 6 months after infection, or 1 year after infection from individuals who had recovered from COVID‐19, Schmidt and colleagues demonstrated that the 50% neutralisation titer values were significantly lower when compared to the Wuhan virus,^80^ suggesting that the omicron variant brings a significant risk of neutralising antibody escape from convalescent patients. This concern is supported by the findings reported from other research groups around the world.^81^, ^82^, ^83^, ^84^

## 9 VACCINE EFFICACY AGAINST THE SARS‐CoV‐2 OMICRON VARIANT

Given that omicron variant has numerous spike mutations that are known to be involved in the immune escape, several studies have been conducted using serum samples obtained from individuals who had been vaccinated (fully or fully with an additional “booster” dose) against SARS‐CoV‐2 to assess whether they would be able to neutralise the SARS‐CoV‐2 omicron variant.^18^, ^33^, ^34^, ^35^, ^73^, ^85^, ^86^, ^87^, ^88^, ^89^, ^90^, ^91^, ^92^ A growing body of data has shown that the omicron variant is associated with immune escape from vaccines‐induced immunity, causing a large number of breakthrough SARS‐CoV‐2 infections in vaccinated populations.^89^, ^90^ Meanwhile, a booster using mRNA vaccines elevated virus‐specific antibody levels and potent neutralisation activity against the omicron variant.^33^, ^36^ In the section below, we summarise the key findings from these studies on the effectiveness of COVID‐19 vaccines for the omicron variant. We also discuss the main immunological characteristics against the omicron variant in vaccinated populations.

Evaluating the effects of a heterologous BNT162b2 mRNA vaccine booster on the humoral immunity of individuals that had received two doses of CoronaVac vaccine, Pérez‐Then and colleagues showed that heterologous CoronaVac prime followed by BNT162b2 booster regimen induced elevated virus‐specific antibody levels and potent neutralisation activity against the SARS‐CoV‐2 Wuhan virus and delta variant, while neutralisation of omicron was undetectable in individuals that had received two‐dose doses of CoronaVac vaccine.^36^ Following the BNT162b2 booster, the results revealed a 1.4‐fold increase in neutralisation activity against omicron variant, compared to two doses of mRNA vaccine.^36^ Interestingly, the neutralising antibody titers were reduced by 7.1‐fold and 3.6‐fold for omicron VOC compared to SARS‐CoV‐2 Wuhan virus and delta VOC, respectively.^36^ Similarly, other reports have found a reduction or no detectable neutralising antibody titer against omicron variant when using the Coronavac vaccine.^35^, ^93^ In summary, these findings suggest that countries primarily using CoronaVac vaccines should consider mRNA vaccine boosters in response to the spread of omicron variant and to combat the impact of further emerging variants.

Recently, Rössler and colleagues evaluated the effectiveness of some COVID‐19 vaccines (mRNA‐1273, ChAdOx1‐S and BNT162b2) against the omicron variant.^94^ They used serum samples collected from individuals who had been infected with the B.1.1.7 (alpha), B.1.351 (beta), or B.1.617.2 (delta) variant of SARS‐CoV‐2 and from individuals who had received two doses of the mRNA‐1273 vaccine (Moderna), the ChAdOx1‐S vaccine (AstraZeneca), or the BNT162b2 vaccine (Pfizer–BioNTech) or had received heterologous vaccination (i.e., one dose each) with the ChAdOx1‐S and BNT162b2 vaccines.^94^ The results revealed that vaccinated individuals neutralised the omicron variant to a much lesser extent than any other SARS‐CoV‐2 variants (alpha, beta, or delta). It was found that some cross‐neutralisation of the omicron variant persists in specimens obtained from individuals who had received either homologous BNT162b2 vaccination or heterologous ChAdOx1‐S–BNT162b2 vaccination but not in specimens from individuals who had received homologous ChAdOx1‐S vaccination.^94^ In that study, the authors found no neutralising antibodies against the omicron variant in serum samples obtained 4–6 months after receipt of the second dose of the mRNA‐1273 vaccine. However, they pointed out that the interval between receipt of the second dose and sample collection in this specific group of vaccinated individuals was longer than for the other vaccination‐regimen groups.^94^

A test‐negative case‐control study evaluated the association between three doses of mRNA COVID‐19 vaccine and symptomatic infection caused by the SARS‐CoV‐2 omicron and delta variants.^86^ Analysing 70,155 tests from symptomatic adults, it was found that individuals who had received three doses of mRNA COVID‐19 vaccine were associated with protection against both the omicron and delta VOCs.^86^ These data suggest that a third dose of the mRNA vaccine increases the vaccine's protective efficacy associated with protection against both the omicron and delta SARS‐CoV‐2 variants.^86^ In a similar report, Lee and colleagues showed that previous infection in octogenarians followed by two doses of BNT162b2 about 1.5 years later resulted in a strong neutralisation based on an ACE2 binding inhibition assay against omicron variant, when compared to persons who had only received two BNT162b2 doses.^95^ In support of these findings, another report measured the neutralisation potency of the serum from 88 mRNA‐1273 (two doses), 111 BNT162b (two doses), and 40 Ad26.COV2.S (one dose) vaccine recipients against SARS‐CoV‐2‐Wuhan virus, delta, and omicron SARS‐CoV‐2 pseudoviruses.^33^ The results demonstrated that neutralising antibodies against the omicron variant were undetectable in most vaccinees.^33^ However, individuals boosted (third dose) with mRNA vaccines demonstrated potent neutralisation of the omicron variant, suggesting that an additional “booster” dose of mRNA vaccine increases breadth and cross‐reactivity of neutralising antibody response among COVID‐19 patients.^33^ These data and the results reported by other research groups underscore the importance of continuing to administer an additional booster dose in the human population.^33^, ^86^, ^88^, ^96^, ^97^, ^98^, ^99^, ^100^, ^101^, ^102^, ^103^, ^104^, ^105^ Despite the improved neutralisation of omicron with additional booster doses, the development of omicron‐specific vaccines should be considered as the variant has become prevalent in most countries.

Another important gap in omicron research is in the assessment of the impact of vaccines on immunocompromised patients. Since the beginning of the pandemic, several studies have shown that immunocompromised patients with COVID‐19 have higher comorbidities, higher risk for prolonged infection, greater levels of inflammatory markers at diagnosis, and higher rates of intensive care admission, and mortality, especially individuals with cancer and who did organ transplants.^106^, ^107^ A recent report provided important insights about the response to omicron in vaccinated individuals with cancer.^108^ Analysing 199 patients with cancer, 115 (58%) of whom had solid tumours and 84 (42%) with blood cancers, all of whom received a third dose of BNT162b2 or two doses of either BNT162b2 (33%) or ChAdOx1 (67%), it was found that most of the individuals with cancer lacked detectable neutralising antibodies against omicron following two vaccine doses, independent of the vaccine type. Meanwhile, with a third dose of BNT162b2, the results revealed a significant increase in neutralising antibodies titers against omicron.^108^ With the possibility that SARS‐CoV‐2 may become endemic,^109^ it will be important to understand the potential risk that VOCs pose to immunocompromised patients.

**FIGURE 3:**
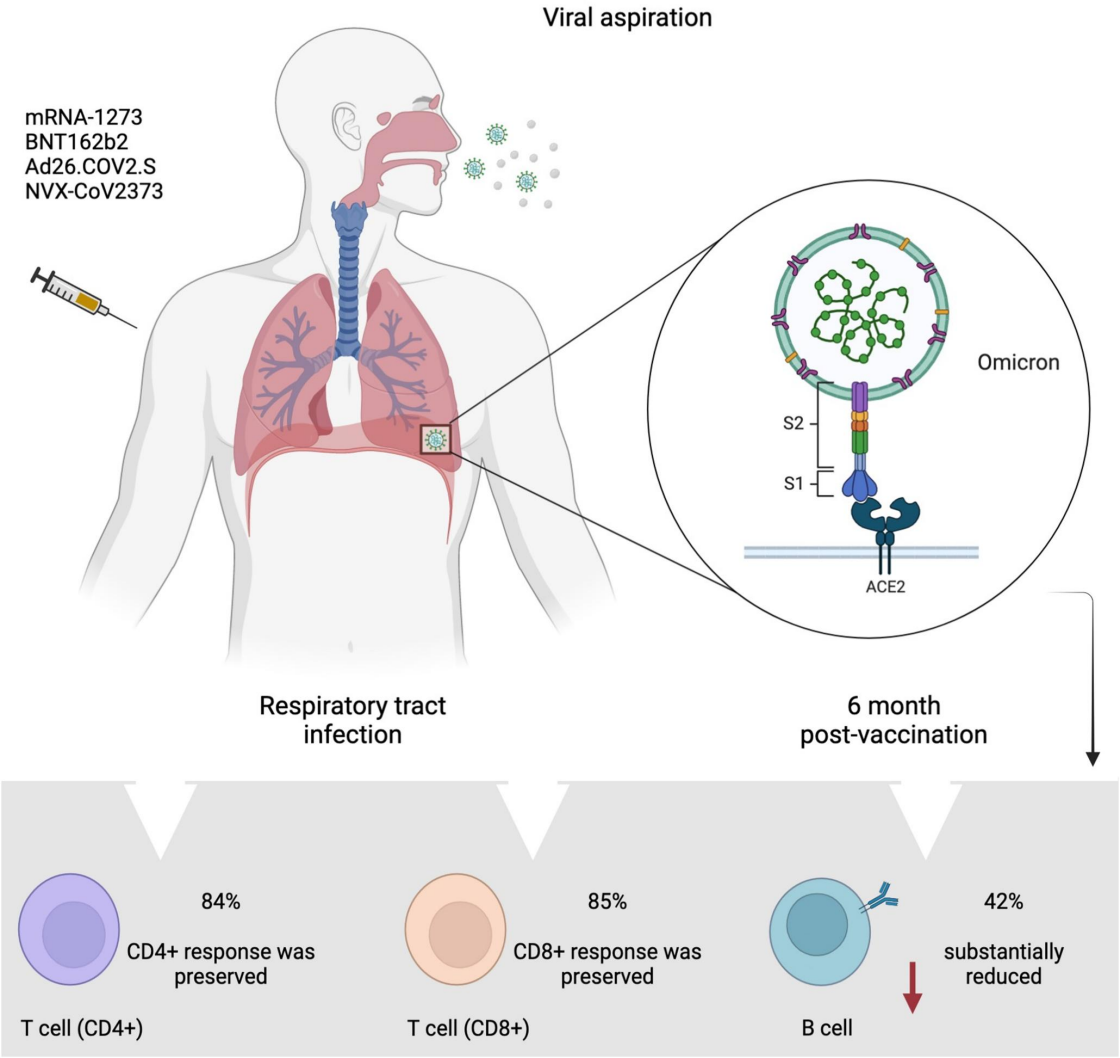
**SARS‐CoV‐2 infection in the respiratory tract and immune response induced by different vaccine platforms against omicron variant.** Analysing specimens obtained from individuals ∼6 months post‐vaccination, it was found that T cell responses are preserved, while significant decreases are observed for memory B cell response. Data used is this figure was obtained from Tarke *et al.* ^114^ ACE2: angiotensin‐converting enzyme 2. The figure was created using Biorender.com

In the context of COVID‐19 vaccination and the emergence of numerous SARS‐CoV‐2 variants, several knowledge gaps remain to be addressed in terms of our understanding in relation to T (CD4+ and CD8+) and B cell immune reactivity. In response, recent studies have been focussed on elucidating immunological features against the omicron variant in vaccinated populations.^100^, ^110^, ^111^, ^112^, ^113^ In one of the earliest reports, Tarke and colleagues evaluated the immune response induced by different vaccine platforms currently used in the human population (mRNA‐1273, BNT162b2, Ad26.COV2.S and NVX‐CoV2373) against several SARS‐CoV‐2 variants including: alpha (B.1.1.7), beta (B.1.351), gamma (P.1), delta (B.1.617.2), omicron (B.1.1.529), kappa (B.1.617.1), lambda (C.37), mu (B.1.621), B.1.1.519, and R.1.^114^ In individuals ∼6 months post‐vaccination with two doses, it was found that T cell responses (84% ‐ CD4+ and 85% ‐ CD8+) were preserved across all COVID‐19 vaccine platforms against the omicron variant. In contrast, significant overall decreases were observed for memory B cell response (42%) when compared to other previous variants, suggesting a preservation of the majority of T cell responses, which may play an important role as second‐level defenses against the omicron and other SARS‐CoV‐2 variants (Figure 3).^114^ Similarly, these outcomes corroborate with recent findings reported by other research teams, suggesting that current COVID‐19 vaccines demonstrate robust protection and most vaccinated individuals retain T‐cell immunity to the SARS‐CoV‐2 omicron variant. This has the potential of balancing the lack of neutralising antibodies, and importantly, preventing or limiting the risk of more severe disease or even death in COVID‐19 patients.^85^, ^100^, ^111^ Within the same perspective, a recent report investigated the memory B cell repertoire in a longitudinal cohort of 42 individuals who had received 3 mRNA vaccine (mRNA‐1273 or BNT162b2) doses.^115^ Following one month after the third dose, the authors revealed that a booster with an mRNA vaccine was accompanied by an increase and evolution of anti‐receptor binding domain‐specific memory B cells, suggesting that these individuals have a diverse memory B cell repertoire that can respond rapidly and produce antibodies capable of clearing VOCs infection such as omicron.^115^

With the emergence of new subvariants of omicron (BA.1.1, BA.2, and BA.3.), recent studies have evaluated the sensitivity to neutralisation by antibodies induced by infection and vaccination using pseudoviruses as a model study. It was found that all currently circulating omicron subvariants evade neutralisation by vaccine‐induced antibodies with comparable high efficiency, suggesting that increased antibody evasion does not represent the main reason for the current dissemination of BA.2 in many countries around the world.^116^

## 10 FINAL CONSIDERATIONS AND PUBLIC HEALTH PERSPECTIVES

The widespread transmission of the SARS‐CoV‐2 omicron variant has been a tremendous challenge for pandemic control, suggesting that we need to reconsider aspects of the virus and disease that had been previously thought to be established. Similarly, our once potent vaccines need to be re‐positioned to address the high mutation rates observed in omicron.^117^ A glimpse of life with endemic SARS‐CoV‐2 may be gained if we examine the well‐known infection characteristics of other respiratory viruses, such as influenza, under conditions in and outside pandemics.^117^ The revaccination of the influenza vaccine has become a recommended annual practice to combat both waning immunity and the appearance of new variants of the virus. After approximately 2 years of the pandemic, two relevant questions arise and remain to be answered. These questions are: (1) Like the flu, will COVID‐19 become a seasonal disease?^109^ and (2) As with influenza, should COVID‐19 vaccines be constantly monitored and vaccine composition updated globally?

The answers appear to be yes to both questions. We will likely require the continued use of vaccines to reduce the incidence of severe illness, hospitalisation and death, even if milder cases still occur at a low frequency. The future timing and composition of booster vaccine doses will need to be determined through experimental, observational and clinical trials as the COVID‐19 pandemic evolves.^117^ Moreover, the non‐pharmaceutical interventions established by the CDC and WHO, such the use of masks, social distancing and avoiding closed spaces, will need to be maintained worldwide, at least for now. Clinical practices like mass testing to detect SARS‐CoV‐2, and isolation of laboratory‐confirmed patients will also likely be required to stay in place. We will need to learn to live with COVID‐19, just as we have learnt to live with flu, with the hope that SARS‐CoV‐2 infection will pose less danger over time.

## AUTHOR CONTRIBUTION

Severino Jefferson Ribeiro da Silva, Alain Kohl, Lindomar Pena and Keith Pardee conceived the work. Severino Jefferson Ribeiro da Silva wrote the original draft. Severino Jefferson Ribeiro da Silva, Alain Kohl, Lindomar Pena and Keith Pardee reviewed the final manuscript. Severino Jefferson Ribeiro da Silva and Keith Pardee supervised the work. All authors critically revised the manuscript and approved the final version of the submitted manuscript.

## CONFLICT OF INTEREST

No conflict of interest declared.

## Data Availability

Data sharing not applicable to this article as no datasets were generated or analysed during the current study.

## References

[1] Zhou P , Yang XL , Wang XG , et al. A pneumonia outbreak associated with a new coronavirus of probable bat origin. Nature. 2020;579(7798):270‐273. ISSN 1476‐4687. https://www.ncbi.nlm.nih.gov/pubmed/32015507 32015507 10.1038/s41586-020-2012-7 PMC7095418

[2] Zhu N , Zhang D , Wang W , et al. A novel coronavirus from patients with pneumonia in China, 2019. N Engl J Med. 2020;382(8):727‐733. ISSN 1533‐4406. https://www.ncbi.nlm.nih.gov/pubmed/31978945 31978945 10.1056/NEJMoa2001017 PMC7092803

[3] Zhong NS , Zheng B , Li Y , et al. Epidemiology and cause of severe acute respiratory syndrome (SARS) in Guangdong, People's Republic of China, in February, 2003. Lancet. 2003;362(9393):1353‐1358. ISSN 1474‐547X. https://www.ncbi.nlm.nih.gov/pubmed/14585636 14585636 10.1016/S0140-6736(03)14630-2 PMC7112415

[4] Zaki AM , vanBoheemen S , Bestebroer TM . Isolation of a novel coronavirus from a man with pneumonia in Saudi Arabia. N Engl J Med. 2012;367(19):1814‐1820. ISSN 1533‐4406. https://www.ncbi.nlm.nih.gov/pubmed/23075143 23075143 10.1056/NEJMoa1211721

[5] WHO . Coronavirus Disease 2019 (COVID‐19); 2020. Situation Report ‐ 51.

[6] Harvey WT , Carabelli AM , Jackson B , et al. SARS‐CoV‐2 variants, spike mutations and immune escape. Nat Rev Microbiol. 2021;19(7):409‐424. ISSN 1740‐1534. https://www.ncbi.nlm.nih.gov/pubmed/34075212 34075212 10.1038/s41579-021-00573-0 PMC8167834

[7] Silva SJRD , Pena L . Collapse of the public health system and the emergence of new variants during the second wave of the COVID‐19 pandemic in Brazil. One Health. 2021;13:100287. ISSN 2352‐7714. 10.1016/j.onehlt.2021.100287. https://www.ncbi.nlm.nih.gov/pubmed/34222607 34222607 PMC8240439

[8] Faria NR , Mellan TA, Whittaker C, et al. Genomics and epidemiology of the P.1 SARS‐CoV‐2 lineage in Manaus, Brazil. Science. 2021. ISSN 1095‐9203. https://www.ncbi.nlm.nih.gov/pubmed/33853970 10.1126/science.abh2644 PMC8139423 33853970

[9] Naveca FG , Nascimento V, Souza VC, et al. COVID‐19 in Amazonas, Brazil, was driven by the persistence of endemic lineages and P.1 emergence. Nat Med. 2021. ISSN 1546‐170X. https://www.ncbi.nlm.nih.gov/pubmed/34035535 10.1038/s41591-021-01378-7 34035535

[10] Tegally H , Wilkinson E , Giovanetti M , et al. Detection of a SARS‐CoV‐2 variant of concern in South Africa. Nature. 2021;592(7854):438‐443. ISSN 1476‐4687. https://www.ncbi.nlm.nih.gov/pubmed/33690265 33690265 10.1038/s41586-021-03402-9

[11] Davies NG , Abbott S , Barnard RC , et al. Estimated transmissibility and impact of SARS‐CoV‐2 lineage B.1.1.7 in England. Science. 2021;372(6538). ISSN 1095‐9203. 10.1126/science.abg3055. https://www.ncbi.nlm.nih.gov/pubmed/33658326 PMC8128288 33658326

[12] Da Silva SJR , Lima SC , Silva RC . Viral load in COVID‐19 patients: implications for prognosis and vaccine efficacy in the context of emerging SARS‐CoV‐2 variants. Front Med. 2021;8:836826. ISSN 2296‐858X. 10.3389/fmed.2021.836826. https://www.ncbi.nlm.nih.gov/pubmed/35174189 PMC8841511 35174189

[13] Lu R , Zhao X , Li J , et al. Genomic characterisation and epidemiology of 2019 novel coronavirus: implications for virus origins and receptor binding. Lancet. 2020;395(10224):565‐574. ISSN 1474‐547X. https://www.ncbi.nlm.nih.gov/pubmed/32007145 32007145 10.1016/S0140-6736(20)30251-8 PMC7159086

[14] Hoffmann M , Kleiner‐Weber H, Schroeder S, et al. SARS‐CoV‐2 cell entry depends on ACE2 and TMPRSS2 and is blocked by a clinically proven protease inhibitor. Cell. 2020. ISSN 1097‐4172. https://www.ncbi.nlm.nih.gov/pubmed/32142651 10.1016/j.cell.2020.02.052 PMC7102627 32142651

[15] Karim SSA , Karim QA . Omicron SARS‐CoV‐2 variant: a new chapter in the COVID‐19 pandemic. Lancet. 2021;398(10317):2126‐2128. ISSN 1474‐547X. https://www.ncbi.nlm.nih.gov/pubmed/34871545 34871545 10.1016/S0140-6736(21)02758-6 PMC8640673

[16] Dong E , Du H , Gardner L . An interactive web‐based dashboard to track COVID‐19 in real time. Lancet Infect Dis. 2020;20(5):533‐534. ISSN 1474‐4457. https://www.ncbi.nlm.nih.gov/pubmed/32087114 32087114 10.1016/S1473-3099(20)30120-1 PMC7159018

[17] Viana R , Moyo S, Amoako DG, et al. Rapid epidemic expansion of the SARS‐CoV‐2 Omicron variant in southern Africa. Nature. 2022;603(7902):679‐686. ISSN 1476‐4687. https://www.ncbi.nlm.nih.gov/pubmed/35042229 35042229 10.1038/s41586-022-04411-y PMC8942855

[18] Hoffmann M , Kruger N, Schulz S, et al. The Omicron variant is highly resistant against antibody‐mediated neutralization: implications for control of the COVID‐19 pandemic. Cell. 2021. ISSN 1097‐4172. https://www.ncbi.nlm.nih.gov/pubmed/35026151 10.1016/j.cell.2021.12.032 PMC8702401 35026151

[19] Dejnirattisai W , Huo J, Zhou D, et al. Omicron‐B.1.1.529 leads to widespread escape from neutralizing antibody responses. bioRxiv. 2021. https://www.ncbi.nlm.nih.gov/pubmed/34981049 10.1016/j.cell.2021.12.046 PMC8723827 35081335

[20] Han P , Li L, Liu S, et al. Receptor binding and complex structures of human ACE2 to spike RBD from omicron and delta SARS‐CoV‐2. Cell. 2022. ISSN 1097‐4172. https://www.ncbi.nlm.nih.gov/pubmed/35093192 10.1016/j.cell.2022.01.001 PMC8733278 35093192

[21] Shuai H , Chan JFW , Hu B , et al. Attenuated replication and pathogenicity of SARS‐CoV‐2 B.1.1.529 Omicron. Nature. 2022;603(7902):693‐699. 10.1038/s41586-022-04442-5 35062016

[22] Zhao H , Lu L , Peng Z , et al. SARS‐CoV‐2 Omicron variant shows less efficient replication and fusion activity when compared with Delta variant in TMPRSS2‐expressed cells. Emerg Microb Infect. 2022;11(1):277‐283. ISSN 2222‐1751. https://www.ncbi.nlm.nih.gov/pubmed/34951565 10.1080/22221751.2021.2023329 PMC8774049 34951565

[23] Suzuki R , Yamasoba D, Kimura I, et al. Attenuated fusogenicity and pathogenicity of SARS‐CoV‐2 Omicron variant. Nature. 2022. ISSN 1476‐4687. https://www.ncbi.nlm.nih.gov/pubmed/35104835 10.1038/s41586-022-04462-1 PMC8942852 35104835

[24] Meng B , Abdullahi A, Ferreira IATM, et al. Altered TMPRSS2 usage by SARS‐CoV‐2 Omicron impacts tropism and fusogenicity. Nature. 2022. ISSN 1476‐4687. https://www.ncbi.nlm.nih.gov/pubmed/35104837 10.1038/s41586-022-04474-x PMC8942856 35104837

[25] Maslo C , Friedland R , Toubkin M , Laubscher A , Akaloo T , Kama B . Characteristics and outcomes of hospitalized patients in South Africa during the COVID‐19 omicron wave compared with previous waves. JAMA. 2022;327(6):583‐584. ISSN 1538‐3598. https://www.ncbi.nlm.nih.gov/pubmed/34967859 34967859 10.1001/jama.2021.24868 PMC8719272

[26] Nyberg T , Ferguson NM , Nash SG , et al. Comparative analysis of the risks of hospitalisation and death associated with SARS‐CoV‐2 omicron (B.1.1.529) and delta (B.1.617.2) variants in England: a cohort study. Lancet. 2022;399(10332):1303‐1312. ISSN 1474‐547X. https://www.ncbi.nlm.nih.gov/pubmed/35305296 35305296 10.1016/S0140-6736(22)00462-7 PMC8926413

[27] Hirose R , Itho Y, Ikegaya H, et al. Differences in environmental stability among SARS‐CoV‐2 variants of concern: omicron has higher stability. bioRxiv. 2022. 10.1016/j.cmi.2022.05.020 PMC9144845 35640841

[28] Cameroni E , Bowen JE , Rosen LE , et al. Broadly neutralizing antibodies overcome SARS‐CoV‐2 Omicron antigenic shift. Nature. 2021. ISSN 1476‐4687. 10.1038/d41586-021-03825-4. https://www.ncbi.nlm.nih.gov/pubmed/35016195 PMC9531318 35016195

[29] Planas D , Saunders N , Maes P , et al. Considerable escape of SARS‐CoV‐2 Omicron to antibody neutralization. Nature. 2021. ISSN 1476‐4687. 10.1038/d41586-021-03827-2. https://www.ncbi.nlm.nih.gov/pubmed/35016199 35016199

[30] Vanblargan LA , Errico JM , Halfmann PJ , et al. An infectious SARS‐CoV‐2 B.1.1.529 Omicron virus escapes neutralization by therapeutic monoclonal antibodies. Nat Med. 2022:490‐495. ISSN 1546‐170X. 10.1038/s41591-021-01678-y. https://www.ncbi.nlm.nih.gov/pubmed/35046573 35046573 PMC8767531

[31] Cao Y , Wang J , Jian F , et al. Omicron escapes the majority of existing SARS‐CoV‐2 neutralizing antibodies. Nature. 2021. ISSN 1476‐4687. 10.1038/d41586-021-03796-6. https://www.ncbi.nlm.nih.gov/pubmed/35016194 PMC8866119 35016194

[32] He C , He X , Yang J , et al. Spike protein of SARS‐CoV‐2 Omicron (B.1.1.529) variant have a reduced ability to induce the immune response. Signal Transduct Targeted Ther. 2022;7(1):119. ISSN 2059‐3635. 10.1038/s41392-022-00980-6. https://www.ncbi.nlm.nih.gov/pubmed/35397623 PMC8994023 35397623

[33] Garcia‐Beltran WF , Denis KJS, Hoelzemer A, et al. mRNA‐based COVID‐19 vaccine boosters induce neutralizing immunity against SARS‐CoV‐2 Omicron variant. Cell. 2022. ISSN 1097‐4172. https://www.ncbi.nlm.nih.gov/pubmed/34995482 10.1016/j.cell.2021.12.033 PMC8733787 34995482

[34] Dejnirattisai W , Shaw R, Supasa P, et al. Reduced neutralisation of SARS‐CoV‐2 omicron B.1.1.529 variant by post‐immunisation serum. Lancet. 2021. ISSN 1474‐547X. https://www.ncbi.nlm.nih.gov/pubmed/34942101 10.1016/S0140-6736(21)02844-0 PMC8687667 34942101

[35] Lu L , Mok BWY, Chen LL, et al. Neutralization of SARS‐CoV‐2 Omicron variant by sera from BNT162b2 or Coronavac vaccine recipients. Clin Infect Dis. 2021. ISSN 1537‐6591. https://www.ncbi.nlm.nih.gov/pubmed/34915551 10.1093/cid/ciab1041 PMC8754807 34915551

[36] Pérez‐Then E , Lucas C, Monteiro VS, et al. Neutralizing antibodies against the SARS‐CoV‐2 Delta and Omicron variants following heterologous CoronaVac plus BNT162b2 booster vaccination. Nat Med. 2022. ISSN 1546‐170X. https://www.ncbi.nlm.nih.gov/pubmed/35051990 10.1038/s41591-022-01705-6 PMC8938264 35051990

[37] Lusvarghi S , Pollett SD , Neerukonda SN , et al. SARS‐CoV‐2 BA.1 variant is neutralized by vaccine booster‐elicited serum, but evades most convalescent serum and therapeutic antibodies. Sci Transl Med. 2022:eabn8543. ISSN 1946‐6242. 10.1126/scitranslmed.abn8543. https://www.ncbi.nlm.nih.gov/pubmed/35380448 35380448 PMC8995032

[38] Evans JP , Zeng C , Carlin C , et al. Neutralizing antibody responses elicited by SARS‐CoV‐2 mRNA vaccination wane over time and are boosted by breakthrough infection. Sci Transl Med. 2022;14(637):eabn8057. ISSN 1946‐6242. 10.1126/scitranslmed.abn8057. https://www.ncbi.nlm.nih.gov/pubmed/35166573 35166573 PMC8939766

[39] Hoffmann M , Arora P , GroB R , et al. SARS‐CoV‐2 variants B.1.351 and P.1 escape from neutralizing antibodies. Cell. 2021;184(9):2384‐2393.e12. ISSN 1097‐4172. https://www.ncbi.nlm.nih.gov/pubmed/33794143 33794143 10.1016/j.cell.2021.03.036 PMC7980144

[40] Jung C , Kmiec D , Koepke L , et al. Omicron: what makes the latest SARS‐CoV‐2 variant of concern so concerning? J Virol. 2022;96(6):e0207721. ISSN 1098‐5514. 10.1128/jvi.02077-21. https://www.ncbi.nlm.nih.gov/pubmed/35225672 35225672 PMC8941872

[41] Rambaut A , Holmes EC , O’Toole A , et al. A dynamic nomenclature proposal for SARS‐CoV‐2 lineages to assist genomic epidemiology. Nat Microbiol. 2020;5(11):1403‐1407. ISSN 2058‐5276. https://www.ncbi.nlm.nih.gov/pubmed/32669681 32669681 10.1038/s41564-020-0770-5 PMC7610519

[42] Yu J , Collier AY , Rowe M , et al. Neutralization of the SARS‐CoV‐2 omicron BA.1 and BA.2 variants. N Engl J Med. 2022:1579‐1580. ISSN 1533‐4406. 10.1056/nejmc2201849. https://www.ncbi.nlm.nih.gov/pubmed/35294809 35294809 PMC9006770

[43] Iketani S , Liu L , Guo Y , et al. Antibody evasion properties of SARS‐CoV‐2 Omicron sublineages. Nature. 2022:553‐556. ISSN 1476‐4687. 10.1038/s41586-022-04594-4. https://www.ncbi.nlm.nih.gov/pubmed/35240676 35240676 PMC9021018

[44] Bruel T , Hadjadj J , Maes P , et al. Serum neutralization of SARS‐CoV‐2 Omicron sublineages BA.1 and BA.2 in patients receiving monoclonal antibodies. Nat Med. 2022. ISSN 1546‐170X. 10.1038/s41591-022-01792-5. https://www.ncbi.nlm.nih.gov/pubmed/35322239 35322239

[45] WORLD HEALTH ORGANIZATION, W. H . Update on Omicron; 2022.

[46] Chen J , Wang R , Gilby NB . Omicron variant (B.1.1.529): infectivity, vaccine breakthrough, and antibody resistance. J Chem Inf Model. 2022:412‐422. ISSN 1549‐960X. 10.1021/acs.jcim.1c01451. https://www.ncbi.nlm.nih.gov/pubmed/34989238 34989238 PMC8751645

[47] Sofonea M , et al. From Delta to Omicron: analysing the SARS‐CoV‐2 epidemic in France using variant‐specific screening tests (September 1 to December 18, 2021). medRxiv. 2022.

[48] Yang W , Shaman J . SARS‐CoV‐2 transmission dynamics in South Africa and epidemiological characteristics of the Omicron variant. medRxiv. 2021. https://www.ncbi.nlm.nih.gov/pubmed/34981071

[49] Hui KPY , Ho JCW , Cheung M , et al. SARS‐CoV‐2 Omicron variant replication in human bronchus and lung ex vivo. Nature. 2022:715‐720. ISSN 1476‐4687. 10.1038/s41586-022-04479-6. https://www.ncbi.nlm.nih.gov/pubmed/35104836 35104836

[50] Peacock T , Brown JC, Zhou J, et al. The SARS‐CoV‐2 variant, Omicron, shows rapid replication in human primary nasal epithelial cultures and efficiently uses the endosomal route of entry. bioRxiv. 2021.

[51] Wu L , Zhou L , Mo M , et al. SARS‐CoV‐2 Omicron RBD shows weaker binding affinity than the currently dominant Delta variant to human ACE2. Signal Transduct Targeted Ther. 2022;7(1):8. ISSN 2059‐3635. 10.1038/s41392-021-00863-2. https://www.ncbi.nlm.nih.gov/pubmed/34987150 PMC8727475 34987150

[52] Lupala CS , Ye Y , Chen H . Mutations on RBD of SARS‐CoV‐2 Omicron variant result in stronger binding to human ACE2 receptor. Biochem Biophys Res Commun. 2022;590:34‐41. ISSN 1090‐2104. https://www.ncbi.nlm.nih.gov/pubmed/34968782 34968782 10.1016/j.bbrc.2021.12.079 PMC8702632

[53] Cui Z , Liu P , Wang N , et al. Structural and functional characterizations of infectivity and immune evasion of SARS‐CoV‐2 Omicron. Cell. 2022:860‐871.e13. ISSN 1097‐4172. 10.1016/j.cell.2022.01.019. https://www.ncbi.nlm.nih.gov/pubmed/35120603 35120603 PMC8786603

[54] Ortega JT , Jastrzebska B , Rangel HR . Omicron SARS‐CoV‐2 variant spike protein shows an increased affinity to the human ACE2 receptor: an in silico analysis. Pathogens. 2021;11(1). ISSN 2076‐0817. 10.3390/pathogens11010045. https://www.ncbi.nlm.nih.gov/pubmed/35055993 PMC8779645 35055993

[55] Mannar D , Saville JW , Zhu X , et al. SARS‐CoV‐2 Omicron variant: antibody evasion and cryo‐EM structure of spike protein‐ACE2 complex. Science. 2022:eabn7760‐764. ISSN 1095‐9203. 10.1126/science.abn7760. https://www.ncbi.nlm.nih.gov/pubmed/35050643 PMC9799367 35050643

[56] Saito A , Irie T , Suzuki R , et al. Enhanced fusogenicity and pathogenicity of SARS‐CoV‐2 Delta P681R mutation. Nature. 2021:300‐306. ISSN 1476‐4687. 10.1038/s41586-021-04266-9. https://www.ncbi.nlm.nih.gov/pubmed/34823256 PMC8828475 34823256

[57] Mlcochova P , Kemp SA, Dhar MS, et al. SARS‐CoV‐2 B.1.617.2 Delta variant replication and immune evasion. Nature. 2021;599(7883):114‐119. ISSN 1476‐4687. https://www.ncbi.nlm.nih.gov/pubmed/34488225 34488225 10.1038/s41586-021-03944-y PMC8566220

[58] Menni C , Valdes AM , Polidori L , et al. Symptom prevalence, duration, and risk of hospital admission in individuals infected with SARS‐CoV‐2 during periods of omicron and delta variant dominance: a prospective observational study from the ZOE COVID Study. Lancet. 2022:1618‐1624. ISSN 1474‐547X. 10.1016/s0140-6736(22)00327-0. https://www.ncbi.nlm.nih.gov/pubmed/35397851 35397851 PMC8989396

[59] Team CC‐R . SARS‐CoV‐2 B.1.1.529 (omicron) variant ‐ United States, december 1‐8, 2021. MMWR Morb Mortal Wkly Rep. 2021;70(50):1731‐1734. ISSN 1545‐861X. https://www.ncbi.nlm.nih.gov/pubmed/34914670 34914670 10.15585/mmwr.mm7050e1 PMC8675659

[60] Wang L , Berger NA, Kaelber DC, Davis PB, Volkow ND, Xu R. Comparison of outcomes from COVID infection in pediatric and adult patients before and after the emergence of Omicron. medRxiv. 2022.

[61] Wolter N , Jassat W , Walaza S , et al. Early assessment of the clinical severity of the SARS‐CoV‐2 omicron variant in South Africa: a data linkage study. Lancet. 2022:437‐446. ISSN 1474‐547X. 10.1016/s0140-6736(22)00017-4. https://www.ncbi.nlm.nih.gov/pubmed/35065011 35065011 PMC8769664

[62] Bal A , Destras G , Gaymard A , et al. Two‐step strategy for the identification of SARS‐CoV‐2 variant of concern 202012/01 and other variants with spike deletion H69‐V70, France, August to December 2020. Euro Surveill. 2021;26(3). ISSN 1560‐7917. 10.2807/1560-7917.es.2021.26.3.2100008. https://www.ncbi.nlm.nih.gov/pubmed/33478625 PMC7848679 33478625

[63] Li A , Maier L, Carter M, Guan TH. Omicron and S‐gene target failure cases in the highest COVID‐19 case rate region in Canada‐December 2021. J Med Virol. 2021. ISSN 1096‐9071. https://www.ncbi.nlm.nih.gov/pubmed/34964500 10.1002/jmv.27562 PMC9015418 34964500

[64] Halfmann PJ , Lida S, Iwatsuki‐Horimoto K, et al. SARS‐CoV‐2 Omicron virus causes attenuated disease in mice and hamsters. Nature. 2022. ISSN 1476‐4687. https://www.ncbi.nlm.nih.gov/pubmed/35062015 10.1038/s41586-022-04441-6 PMC8942849 35062015

[65] Mohandas S , Yadav PD , Sapkal G , et al. Pathogenicity of SARS‐CoV‐2 Omicron (R346K) variant in Syrian hamsters and its cross‐neutralization with different variants of concern. EBioMedicine. 2022;79:103997. ISSN 2352‐3964. 10.1016/j.ebiom.2022.103997. https://www.ncbi.nlm.nih.gov/pubmed/35405385 35405385 PMC8993158

[66] Barnard RC , Davies N, Pearson CAB, Jit M, Edmunds WJ. Modelling the Potential Consequences of the Omicron SARS‐CoV‐2 Variant in England. CMMID; 2021.

[67] Zhang R , Li Y , Zhang AL . Identifying airborne transmission as the dominant route for the spread of COVID‐19. Proc Natl Acad Sci U. S. A. 2020;117(26):14857‐14863. ISSN 1091‐6490. https://www.ncbi.nlm.nih.gov/pubmed/32527856 32527856 10.1073/pnas.2009637117 PMC7334447

[68] Falahi S , Kenarkoohi A . Transmission routes for SARS‐CoV‐2 infection: review of evidence. New Microbes New Infect. 2020;38:100778. ISSN 2052‐2975. 10.1016/j.nmni.2020.100778. https://www.ncbi.nlm.nih.gov/pubmed/33042554 33042554 PMC7537649

[69] Silva SJRD , Nascimento JCF , Santos Reis WPM , et al. Widespread contamination of SARS‐CoV‐2 on highly touched surfaces in Brazil during the second wave of the COVID‐19 pandemic. Environ Microbiol. 2021:7382‐7395. ISSN 1462‐2920. 10.1111/1462-2920.15855. https://www.ncbi.nlm.nih.gov/pubmed/34863010 34863010 PMC9303906

[70] Taylor PC , Adams AC , Hufford MM , dela Torre I , Winthrop K , Gottlieb RL . Neutralizing monoclonal antibodies for treatment of COVID‐19. Nat Rev Immunol. 2021;21(6):382‐393. ISSN 1474‐1741. https://www.ncbi.nlm.nih.gov/pubmed/33875867 33875867 10.1038/s41577-021-00542-x PMC8054133

[71] Lusvarghi S , Pollett SD, Neerukonda SN, et al. SARS‐CoV‐2 Omicron neutralization by therapeutic antibodies, convalescent sera, and post‐mRNA vaccine booster. bioRxiv. 2021. https://www.ncbi.nlm.nih.gov/pubmed/34981057

[72] Takashita E , Kinoshita N , Yamayoshi S , et al. Efficacy of antibodies and antiviral drugs against covid‐19 omicron variant. N Engl J Med. 2022:995‐998. ISSN 1533‐4406. 10.1056/nejmc2119407. https://www.ncbi.nlm.nih.gov/pubmed/35081300 35081300 PMC8809508

[73] Liu L , Iketani S , Guo Y , et al. Striking antibody evasion manifested by the omicron variant of SARS‐CoV‐2. Nature. 2021. ISSN 1476‐4687. 10.1038/d41586-021-03826-3. https://www.ncbi.nlm.nih.gov/pubmed/35016198 35016198

[74] FDA . Coronavirus (COVID‐19) Update: FDA Limits Use of Certain Monoclonal Antibodies to Treat COVID‐19 Due to the Omicron Variant; 2022.

[75] Mccallum M , Czudnochowski N , Rosen LE , et al. Structural basis of SARS‐CoV‐2 Omicron immune evasion and receptor engagement. Science. 2022:eabn8652‐868. ISSN 1095‐9203. 10.1126/science.abn8652. https://www.ncbi.nlm.nih.gov/pubmed/35076256 PMC9427005 35076256

[76] Yin W , Xu Y , Xu P , et al. Structures of the Omicron Spike trimer with ACE2 and an anti‐Omicron antibody. Science. 2022:1048‐1053. ISSN 1095‐9203. 10.1126/science.abn8863. https://www.ncbi.nlm.nih.gov/pubmed/35133176 PMC8939775 35133176

[77] Vangeel L , Chiu W , De Jonghe S , et al. Remdesivir, Molnupiravir and Nirmatrelvir remain active against SARS‐CoV‐2 Omicron and other variants of concern. Antiviral Res. 2022;198:105252. ISSN 1872‐9096. 10.1016/j.antiviral.2022.105252. https://www.ncbi.nlm.nih.gov/pubmed/35085683 35085683 PMC8785409

[78] Li P , Wang Y , Lavrijsen M , et al. SARS‐CoV‐2 Omicron variant is highly sensitive to molnupiravir, nirmatrelvir, and the combination. Cell Res. 2022;32(3):322‐324. ISSN 1748‐7838. https://www.ncbi.nlm.nih.gov/pubmed/35058606 35058606 10.1038/s41422-022-00618-w PMC8771185

[79] Dupont L , Snell LB , Graham C , et al. Neutralizing antibody activity in convalescent sera from infection in humans with SARS‐CoV‐2 and variants of concern. Nat Microbiol. 2021;6(11):1433‐1442. ISSN 2058‐5276. https://www.ncbi.nlm.nih.gov/pubmed/34654917 34654917 10.1038/s41564-021-00974-0 PMC8556155

[80] Schmidt F , Mueckach F, Weisblum Y, et al. Plasma neutralization of the SARS‐CoV‐2 omicron variant. N Engl J Med. 2021. ISSN 1533‐4406. https://www.ncbi.nlm.nih.gov/pubmed/35030645 10.1056/NEJMc2119641 PMC8757565 35030645

[81] Wang Y , Ma Y , Xu Y , et al. Resistance of SARS‐CoV‐2 Omicron variant to convalescent and CoronaVac vaccine plasma. Emerg Microb Infect. 2022;11(1):424‐427. ISSN 2222‐1751. https://www.ncbi.nlm.nih.gov/pubmed/35001836 10.1080/22221751.2022.2027219 PMC8803103 35001836

[82] Carreño JM , Alshammary H , Tcheou J , et al. Activity of convalescent and vaccine serum against SARS‐CoV‐2 Omicron. Nature. 2022;602(7898):682‐688. ISSN 1476‐4687. https://www.ncbi.nlm.nih.gov/pubmed/35016197 35016197 10.1038/s41586-022-04399-5

[83] Zhang X , Wu S , Wu B , et al. SARS‐CoV‐2 Omicron strain exhibits potent capabilities for immune evasion and viral entrance. Signal Transduct Targeted Ther. 2021;6(1):430. ISSN 2059‐3635. 10.1038/s41392-021-00852-5. https://www.ncbi.nlm.nih.gov/pubmed/34921135 PMC8678971 34921135

[84] Zou J , Xia H , Xie X , et al. Neutralization against Omicron SARS‐CoV‐2 from previous non‐Omicron infection. Nat Commun. 2022;13(1):852. ISSN 2041‐1723. 10.1038/s41467-022-28544-w. https://www.ncbi.nlm.nih.gov/pubmed/35140233 35140233 PMC8828871

[85] Collie S , Champion J, Moultrie H, Bekker LG, Gray G. Effectiveness of BNT162b2 vaccine against omicron variant in South Africa. N Engl J Med. 2021. ISSN 1533‐4406. https://www.ncbi.nlm.nih.gov/pubmed/34965358 10.1056/NEJMc2119270 PMC8757569 34965358

[86] Accorsi EK , Britton A , Fleming‐Dutra KE , et al. Association between 3 doses of mRNA COVID‐19 vaccine and symptomatic infection caused by the SARS‐CoV‐2 omicron and delta variants. JAMA. 2022:639. ISSN 1538‐3598. 10.1001/jama.2022.0470. https://www.ncbi.nlm.nih.gov/pubmed/35060999 35060999 PMC8848203

[87] Cele S , Jackson L , Khoury DS , et al. Omicron extensively but incompletely escapes Pfizer BNT162b2 neutralization. Nature. 2021. ISSN 1476‐4687. 10.1038/d41586-021-03824-5. https://www.ncbi.nlm.nih.gov/pubmed/35016196 PMC8866126 35016196

[88] Muik A , Lui BG , Wallisch AK , et al. Neutralization of SARS‐CoV‐2 Omicron by BNT162b2 mRNA vaccine‐elicited human sera. Science. 2022:eabn7591‐680. ISSN 1095‐9203. 10.1126/science.abn7591. https://www.ncbi.nlm.nih.gov/pubmed/35040667 PMC9836206 35040667

[89] Pulliam J , Schalkwyk CV, Govender N, et al. Increased risk of SARS‐CoV‐2 reinfection associated with emergence of the Omicron variant in South Africa. medRxiv. 2021. 10.1126/science.abn4947 PMC8995029 35289632

[90] Kuhlmann C , Mayer CK , Claassen M , et al. Breakthrough infections with SARS‐CoV‐2 omicron despite mRNA vaccine booster dose. Lancet. 2022;399(10325):625‐626. 10.1016/s0140-6736(22)00090-3 35063123 PMC8765759

[91] Kitchin D , Richardson SI , van der Mescht MA , et al. Ad26.COV2.S breakthrough infections induce high titers of neutralizing antibodies against Omicron and other SARS‐CoV‐2 variants of concern. Cell Rep Med. 2022;3(3):100535. 10.1016/j.xcrm.2022.100535 35474744 PMC8828412

[92] Edara VV , Manning KE , Ellis M , et al. mRNA‐1273 and BNT162b2 mRNA vaccines have reduced neutralizing activity against the SARS‐CoV‐2 omicron variant. Cell Rep Med. 2022;3(2):100529. ISSN 2666‐3791. 10.1016/j.xcrm.2022.100529. https://www.ncbi.nlm.nih.gov/pubmed/35233550 35233550 PMC8784612

[93] Cheng SMS , Mok CKP, Leung YWY, et al. Neutralizing antibodies against the SARS‐CoV‐2 Omicron variant following homologous and heterologous CoronaVac or BNT162b2 vaccination. Nat Med. 2022. ISSN 1546‐170X. https://www.ncbi.nlm.nih.gov/pubmed/35051989 10.1038/s41591-022-01704-7 PMC8940714 35051989

[94] Rössler A , Riepler L , Bante D . SARS‐CoV‐2 omicron variant neutralization in serum from vaccinated and convalescent persons. N Engl J Med. 2022:698‐700. ISSN 1533‐4406. 10.1056/nejmc2119236. https://www.ncbi.nlm.nih.gov/pubmed/35021005 35021005 PMC8781314

[95] Lee HK , Knabl L , Moliva JI , et al. mRNA vaccination in octogenarians 15 and 20 months after recovery from COVID‐19 elicits robust immune and antibody responses that include Omicron. Cell Rep. 2022;39(2):110680. ISSN 2211‐1247. 10.1016/j.celrep.2022.110680. https://www.ncbi.nlm.nih.gov/pubmed/35395191 35395191 PMC8947943

[96] Thompson MG , Natarajan K , Irving SA , et al. Effectiveness of a third dose of mRNA vaccines against COVID‐19‐associated emergency department and urgent care encounters and hospitalizations among adults during periods of delta and omicron variant predominance ‐ VISION network, 10 states, August 2021‐January 2022. MMWR Morb Mortal Wkly Rep. 2022;71(4):139‐145. ISSN 1545‐861X. https://www.ncbi.nlm.nih.gov/pubmed/35085224 35085224 10.15585/mmwr.mm7104e3 PMC9351525

[97] Gruell H , Vanshylla K , Tober‐Lau P , et al. mRNA booster immunization elicits potent neutralizing serum activity against the SARS‐CoV‐2 Omicron variant. Nat Med. 2022:477‐480. ISSN 1546‐170X. 10.1038/s41591-021-01676-0. https://www.ncbi.nlm.nih.gov/pubmed/35046572 35046572 PMC8767537

[98] Ai J , Zhang H , Zhang Y , et al. Omicron variant showed lower neutralizing sensitivity than other SARS‐CoV‐2 variants to immune sera elicited by vaccines after boost. Emerg Microb Infect. 2022;11(1):337‐343. ISSN 2222‐1751. https://www.ncbi.nlm.nih.gov/pubmed/34935594 10.1080/22221751.2021.2022440 PMC8788341 34935594

[99] Elliot P , Bodinier B , Eales O , et al. Rapid increase in omicron infections in England during december 2021: REACT‐1 study. Science. 2022;375(6587):1406‐1411. 10.1126/science.abn8347 35133177 PMC8939772

[100] Geurtsvankessel CH , Geers D, Schmitz KS, et al. Divergent SARS CoV‐2 Omicron‐reactive T‐ and B cell responses in COVID‐19 vaccine recipients. Sci Immunol. 2022:eabo2202. ISSN 2470‐9468. https://www.ncbi.nlm.nih.gov/pubmed/35113647 35113647 10.1126/sciimmunol.abo2202 PMC8939771

[101] Walls AC , Sprouse KR , Bowen JE , et al. SARS‐CoV‐2 breakthrough infections elicit potent, broad, and durable neutralizing antibody responses. Cell. 2022:872‐880.e3. ISSN 1097‐4172. 10.1016/j.cell.2022.01.011. https://www.ncbi.nlm.nih.gov/pubmed/35123650 35123650 PMC8769922

[102] Ariën KK , Heyndrickx L , Michiels J , et al. Three doses of BNT162b2 vaccine confer neutralising antibody capacity against the SARS‐CoV‐2 Omicron variant. NPJ Vaccines. 2022;7(1):35. ISSN 2059‐0105. 10.1038/s41541-022-00459-z. https://www.ncbi.nlm.nih.gov/pubmed/35260578 35260578 PMC8904765

[103] Tseng HF , Ackerson BK , Luo Y , et al. Effectiveness of mRNA‐1273 against SARS‐CoV‐2 omicron and delta variants. Nat Med. 2022:1063‐1071. ISSN 1546‐170X. 10.1038/s41591-022-01753-y. https://www.ncbi.nlm.nih.gov/pubmed/35189624 35189624 PMC9117141

[104] Nemet I , Kliker L , Lustig Y , et al. Third BNT162b2 vaccination neutralization of SARS‐CoV‐2 omicron infection. N Engl J Med. 2022;386(5):492‐494. ISSN 1533‐4406. https://www.ncbi.nlm.nih.gov/pubmed/34965337 34965337 10.1056/NEJMc2119358 PMC8823651

[105] Plumb ID , Feldstein LR , Barkley E , et al. Effectiveness of COVID‐19 mRNA vaccination in preventing COVID‐19‐associated hospitalization among adults with previous SARS‐CoV‐2 infection ‐ United States, June 2021‐February 2022. MMWR Morb Mortal Wkly Rep. 2022;71(15):549‐555. ISSN 1545‐861X. https://www.ncbi.nlm.nih.gov/pubmed/35421077 35421077 10.15585/mmwr.mm7115e2 PMC9020856

[106] Belsky JA , Tullius BP , Lamb MG , Sayegh R , Stanek JR , Auletta JJ . COVID‐19 in immunocompromised patients: a systematic review of cancer, hematopoietic cell and solid organ transplant patients. J Infect. 2021;82(3):329‐338. ISSN 1532‐2742. https://www.ncbi.nlm.nih.gov/pubmed/33549624 33549624 10.1016/j.jinf.2021.01.022 PMC7859698

[107] Corey L , Beyrer C , Cohen MS , Michael NL , Bedford T , Rolland M . SARS‐CoV‐2 variants in patients with Immunosuppression. N Engl J Med. 2021;385(6):562‐566. ISSN 1533‐4406. https://www.ncbi.nlm.nih.gov/pubmed/34347959 34347959 10.1056/NEJMsb2104756 PMC8494465

[108] Fendler A , Shepherd STC, Au L, et al. Omicron neutralising antibodies after third COVID‐19 vaccine dose in patients with cancer. Lancet. 2022. ISSN 1474‐547X. https://www.ncbi.nlm.nih.gov/pubmed/35090602 10.1016/S0140-6736(22)00147-7 PMC8789238 35090602

[109] Phillips N . The coronavirus is here to stay ‐ here's what that means. Nature. 2021;590(7846):382‐384. ISSN 1476‐4687. https://www.ncbi.nlm.nih.gov/pubmed/33594289 33594289 10.1038/d41586-021-00396-2

[110] Kotaki R , Adachi Y , Moriyama S , et al. SARS‐CoV‐2 Omicron‐neutralizing memory B‐cells are elicited by two doses of BNT162b2 mRNA vaccine. Sci Immunol. 2022:8590. ISSN 2470‐9468. 10.1126/sciimmunol.abn8590. https://www.ncbi.nlm.nih.gov/pubmed/35113654 PMC8939773 35113654

[111] Liu J , Chandrashekar A , Sellers D , et al. Vaccines elicit highly conserved cellular immunity to SARS‐CoV‐2 omicron. Nature. 2022:493‐496. ISSN 1476‐4687. 10.1038/s41586-022-04465-y. https://www.ncbi.nlm.nih.gov/pubmed/35102312 PMC8930761 35102312

[112] Keeton R , Tincho MB, Ngomti A, et al. T cell responses to SARS‐CoV‐2 spike cross‐recognize Omicron. Nature. 2022. ISSN 1476‐4687. https://www.ncbi.nlm.nih.gov/pubmed/35102311 10.1038/s41586-022-04460-3 PMC8930768 35102311

[113] Naranbhai V , Nathan A, Kaseke C, et al. T cell reactivity to the SARS‐CoV‐2 Omicron variant is preserved in most but not all prior infected and vaccinated individuals. medRxiv. 2022. https://www.ncbi.nlm.nih.gov/pubmed/35018386

[114] Tarke A , Coelho CH , Zhang Z , et al. SARS‐CoV‐2 vaccination induces immunological T cell memory able to cross‐recognize variants from Alpha to Omicron. Cell. 2022;185(5):847‐859.e11. ISSN 1097‐4172. https://www.ncbi.nlm.nih.gov/pubmed/35139340 35139340 10.1016/j.cell.2022.01.015 PMC8784649

[115] Muecksch F , Wang Z , Cho A , et al. Increased memory B cell potency and breadth after a SARS‐CoV‐2 mRNA boost. Nature. 2022. ISSN 1476‐4687. 10.1038/s41586-022-04778-y. https://www.ncbi.nlm.nih.gov/pubmed/35447027 PMC9259484 35447027

[116] Arora P , Zhang L , Rocha C , et al. Comparable neutralisation evasion of SARS‐CoV‐2 omicron subvariants BA.1, BA.2, and BA.3. Lancet Infect Dis. 2022. 10.1016/s1473-3099(22)00224-9 PMC9005119 35427493

[117] Monto AS . The future of SARS‐CoV‐2 vaccination ‐ lessons from influenza. N Engl J Med. 2021;385(20):1825‐1827. ISSN 1533‐4406. https://www.ncbi.nlm.nih.gov/pubmed/34739199 34739199 10.1056/NEJMp2113403

